# Editorials

**Published:** 1911-10

**Authors:** 


### EDITORIALS

The Business Office of the *Journal-Record* is 1 1-2 to 5 1-2 South Broad Street.
The Editorial Office is 1014-15 Century Building.
Address all Business Communications to *Journal-Record of Medicine*, 1 1-2 to 5 1-2 South Broad Street.
Make remittances payable *to The Journal-Record of Medicine.*

On matters pertaining to the Editorial and Original Communications, address Edgar G. Ballenger, M. D., Atlanta, Ga.
Reprints of Original Articles will be furnished at cost price. Requests for the same should always be made in THE MANUSCRIPT.

We will present, postpaid, on request, to each contributor of an original article, twenty (20) marked copies of *The Journal-Record of Medicine* containing such article.

### PHYSICIANS' RECORDS AND ACCOUNTS.

In recent years physicians are slowly, too slowly, learning to devote more attention to accounts and records and to more or less prompt collection of bills. The advances in medicine and surgery, many of them necessitating expensive apparatus and post-graduate courses, require money. Now it is the physician who collects his bills and equips himself to give his patients modern, scientific treatment who makes his work a pelasure and a success rather than the man who fails to keep abreast the times, fails to give good treatment and thus realizing the poor quality of the service rendered fails to push the collection of his bills and by this vicious circle of circumstances is forced to continue poor work with poor pay.

The best way to break this malign influence is to strive to do a little better work and show a little more personal interest in the patient, keep more accurate records, send bills more promptly, take a few more medical journals and read them, buy little better office equipment—not junk for show, but things of known value, go once a year to some medical center to learn new methods and improved techniques. Your patients will quickly recognize and appreciate your improvement and will pay more readily their bills.

A wholesome sign is the increased attention to the business side of medicine by medical journals, in fact a new journal has just been started in Chicago called "Successful Medicine," which is deovted to the practical business side of medicine.

The time will come some day when the failure to send and collect a bill will be taken as an admission of inferiority of the service rendered.

